# Risk Factors for Mortality in COVID-19 Hospitalized Patients in Piedmont, Italy: Results from the Multicenter, Regional, CORACLE Registry

**DOI:** 10.3390/jcm10091951

**Published:** 2021-05-01

**Authors:** Francesco Giuseppe De Rosa, Annagloria Palazzo, Tiziana Rosso, Nour Shbaklo, Marco Mussa, Lucio Boglione, Enrica Borgogno, Antonella Rossati, Simone Mornese Pinna, Silvia Scabini, Guido Chichino, Silvio Borrè, Valerio Del Bono, Pietro Luigi Garavelli, Diego Barillà, Francesco Cattel, Giovanni Di Perri, Giovannino Ciccone, Tommaso Lupia, Silvia Corcione

**Affiliations:** 1 Department of Medical Sciences, University of Turin, 10126 Turin, Italy; francescogiuseppe.derosa@unito.it (F.G.D.R.); annagloria.palazzo@unito.it (A.P.); nour.shbaklo@edu.unito.it (N.S.); simone.mornesepinna@unito.it (S.M.P.); silvia.scabini@unito.it (S.S.); Giovanni.diperri@unito.it (G.D.P.); silvia.corcione@unito.it (S.C.); 2 Infectious Diseases Unit, Cardinal Massaia Hospital, 14100 Asti, Italy; 3 Unit of Clinical Epidemiology, CPO, AOU “Città della Salute e della Scienza”, 10126 Turin, Italy; tiziana.rosso@cpo.it (T.R.); gciccone@cittadellasalute.to.it (G.C.); 4 Infectious Diseases Unit, Azienda Ospedaliera SS Antonio e Biagio e Cesare Arrigo, 15121 Alessandria, Italy; marco.mussa@ospedale.al.it (M.M.); guido.chichino@ospedale.al.it (G.C.); 5 Department of Translational Medicine, University of Eastern Piedmont, 13100 Novara, Italy; lucio.boglione@uniupo.it; 6 Infectious Diseases Unit, Azienda Ospedaliera S. Croce e Carle, 12100 Cuneo, Italy; borgogno.e@ospedale.cuneo.it (E.B.); delbono.v@ospedale.cuneo.it (V.D.B.); 7 Infectious Diseases Department, University Hospital “Maggiore della Carità”, 28100 Novara, Italy; antonella.rossati@maggioreosp.novara.it (A.R.); infettivi.dir@maggioreosp.novara.it (P.L.G.); 8 Unit of Infectious Diseases, Saint Andrea Hospital, 13100 Vercelli, Italy; silvio.borre@aslvc.piemonte.it; 9 Hospital Pharmacy, Città della Salute e della Scienza, 10126 Turin, Italy; dbarilla@cittadellasalute.to.it (D.B.); fcattel@gmail.com (F.C.); 10 Department of Infectious Diseases, Tufts University School of Medicine, Boston, MA 02109, USA

**Keywords:** COVID-19, pneumonia, mortality, hydroxichloroquine, SARS-CoV-2

## Abstract

Background: CORACLE is a retrospective and prospective, regional multicenter registry, developed to evaluate risk factors for mortality in a cohort of patients admitted with SARS-CoV-2 infection within non-intensive wards. Methods: The primary objective was to estimate the role of several prognostic factors on hospital mortality in terms of adjusted Odds Ratios (aOR) with multivariable logistic regression models. Results: A total of 1538 patients were enrolled; 42% were female, and 58% were >70 years old. Deceased patients were 422 (27%), with a median age of 83 years (IQR (Inter Quartile Range) 76–87). Older age at admission (aOR 1.07 per year, 95%CI 1.06–1.09), diabetes (1.41, 1.02–1.94), cardiovascular disease (1.79, 1.31–2.44), immunosuppression (1.65, 1.04–2.62), estimated glomerular filtration rate (eGFR) <30 mL/min/1.73 m^2^ (3.53, 2.26–5.51), higher C-reactive protein values and a decreased PaO_2_/FiO_2_ ratio at admission were associated with a higher risk of hospital mortality. Amongst patients still alive on day 7, only hydroxychloroquine (HCQ) treatment was associated with reduced mortality (0.57, 0.36–0.90). Conclusions: Several risk factors were associated with mortality in SARS-CoV-2 positive patients. Although HCQ seems to be the only factor significantly associated with reduced mortality, this result is in contrast with evidence from randomized studies. These results should be interpreted in light of the study limitations.

## 1. Introduction

By the end of 2019, an outbreak of respiratory infection and interstitial pneumonia of unknown origin was detected in Wuhan, Hubei province, China [1,2]. The responsible agent was identified as a virus belonging to the Coronaviridae [3]. The new virus was labeled 2019-nCOV and was subsequently renamed SARS-CoV-2 due to its resemblance with the previous pandemic, which was eventually condensed into COVID-19 [4]. The virus started spreading worldwide and caused, as of 14 April 2021, 137.4 million cases and 2.9 million attributed fatalities around the world [5,6,7]. COVID-19 is mainly a respiratory disease with a variable ratio of asymptomatic to symptomatic patients, extending from moderate to severe and critical cases [8]. This range is due to viral, host or other unknown factors, including the transmission link or the local epidemiology [9].

Systemic manifestations have been described as mainly involving neurological, cardiological, pulmonary, and endocrinological systems [10]. Since no specific therapy has yet been developed, a series of treatments were initially administered, although later were shown to be of questionable efficacy [8]. Italy faced the first wave of infected patients in February 2020 and by 14 April 2021, Italy reported more than 115,000 deaths and over 3.7 million cases, with a case–fatality ratio of almost 3.6% [11,12,13].

A variety of meta-analyses and epidemiological registers have summarized risk factors for mortality in hospital in COVID-19 patients, and currently, consistent evidence suggests that a worse pronouncement is related to age, men and several co-morbidities [14,15,16,17]. Besides the fact that the pregnancy of clinical and biochemical parameters found in COVID-19 patients differs based on average patients’ age, gender and health, Mesas et al. recently provided additional data, including 60 studies, in the course of a comprehensive, systemic literature review with 51,225 patients [18].

The purpose of this study was to examine the effect of epidemiological, clinical and therapeutic variables on hospital mortality in non-critical COVID-19 patients in the Piedmont region of Italy.

## 2. Materials and Methods

The aim of the CORACLE Registry, a retrospective, prospective, regional multicenter protocol, was to collect data regarding non-critically ill COVID-19 inpatients during the COVID-19 outbreak. The CORACLE Registry was promoted by the Piedmont Infectious Diseases Unit Network (PIDUN) and was approved by the centers’ ethics committee. As per the approval, written informed consent was not required due to the observational and largely retrospective, anonymous data collection, following European general data protection regulations (GDPR, n. 2016/679). All of the data were reviewed and cross-checked by a team of medical doctors and data managers with expertise in data collection. The data were analyzed by the Unit of Clinical Epidemiology, City of Health and Science, Turin. Inclusion criteria were hospitalized adult patients with COVID-19 with a laboratory-confirmed diagnosis of SARS-CoV-2 infection detected by Real-Time Protein Chain Reaction (RT-PCR) by nasopharyngeal swabs or bronchoalveolar lavage, when available. Critically ill patients at admission, requiring mechanical ventilation or ICU treatments, were excluded.

The data collected included information on the timing of the disease, signs and symptoms, imaging, laboratory results on admission and treatments administered, taken from electronic and paper medical records. Pneumonia was diagnosed based on radiologic abnormalities (i.e., pulmonary infiltrates, pulmonary consolidations, ground-glass opacities) by chest X-rays or CT scan, when available. The laboratory assessments consisted of a complete blood count, blood chemical analysis, coagulation testing, liver and renal function assessment, C-reactive protein (CRP), procalcitonin, lactate dehydrogenase (LDH), troponin and an arterial-blood gas (ABG) test at admission. The laboratory testing comprised samples taken at baseline and during hospitalization. Fever was defined as a temperature of >37.5 °C. Lymphocytopenia was defined as a lymphocyte count of <1000 cells/mm^3^. Acute Respiratory Distress Syndrome (ARDS) was defined according to Berlin criteria [19] as mild (200 < PaO_2_/FiO_2_ ≤ 300), moderate (100 < PaO_2_/FiO_2_ ≤ 200), or severe (PaO_2_/FiO_2_ ≤ 100) associated with suggestive radiological findings. End-stage renal disease (ESRD) was diagnosed using estimated glomerular filtration rate (eGFR), calculated as an eGFR at admission <15 mL/min/1.73 m^2^.

The Registry’s primary goal was to analyze the demographic characteristics and comorbidities at baseline on hospital mortality on all the study population. The secondary aim was to estimate the efficacy on hospital mortality of steroids, hydroxychloroquine (HCQ), remdesivir (RDV), antiretrovirals (e.g., lopinavir/ritonavir, LPV/r; darunavir/cobicistat, DRV/c; darunavir/r, DRV/r) and targeted therapies (e.g., tocilizumab) in the subgroup of patients who were still alive at day 7. Treatment strategies were selected according to the pharmacological evidence in the literature during the first wave of the pandemic and according to treatment protocols used in Piedmont to which urgent changes have been made on the basis of new scientific evidence. This restriction on the analyzed population was adopted to minimize the risk of survival time bias (treatments were recorded during hospitalization without enough details on starting dates). Lastly, the incidence of ARDS and the percentage of patients undergoing mechanical or non-invasive ventilation during hospitalization was estimated. All analyses were performed using STATA (v. 14) (StataCorp LLC, 4905 Midtown Dr, College Station, TX 77845, United States). Continuous variables were expressed as medians and interquartile ranges or simple ranges, as appropriate. Categorical variables were summarized as counts and percentages. No imputation was made for missing data. Descriptive analyses were conducted using chi-square or Mann–Whitney U test, as appropriate, for categorical and continuous variables, respectively. Logistic regression modeling was used for the multivariable analysis of mortality with crude and adjusted odds ratios (ORs) and their 95% confidence intervals (CIs) were estimated using univariate and multivariate logistic regression models. The adjustment variables were chosen with an a priori selection of the variables considered most interesting. To confirm the analyses on drugs restricted to patients alive on day 7, a propensity score (PS) was calculated and used in the logistic model either as a covariate or as a weight to balance the treated and untreated populations (transforming the PS in an inverse probability treatment weight, IPTW). To determine the robustness of the results on drugs, sensitivity analyses were also performed on the total population and those alive at day 3.

## 3. Results

From 27 February to 15 June 2020, 1552 patients were recorded in the registry; 1538 had complete data on in-hospital mortality and were included in the present analysis. The included patients were from Alessandria Hospital (*n* = 491), followed by Asti (*n* = 422), Turin City of Health and Sciences, Molinette Hospital” (*n* = 314), Vercelli (*n* = 195), Cuneo (*n* = 63) and Novara (*n* = 53).

### 3.1. Demographic Characteristics and Comorbidities

The patients’ demographic and clinical characteristics are shown in Table 1. The median age at admission was 74 years (interquartile range, IQR: 61–83 years) and 42% were females. Of the sample, 76 subjects (7%) actively smoke, 256 (23%) were former smokers and 789 (70%) have never smoked. Most frequent comorbidities were hypertension (49%), cardiovascular disease (32%, including a history of stroke, cardiac failure, and myocardial infarction); diabetes mellitus (21%) and lung disease (13%, mostly chronic obstructive pulmonary disease—COPD). There were 148 (10%) patients with various degrees of immunosuppression: 74 (5%) had active solid cancer and were under active chemotherapy or radiotherapy, 38 (2%) suffered from onco-hematological diseases (i.e., leukemia, lymphomas, and multiple myeloma), 21 (1%) were on courses of immunosuppressive therapies or had a solid organ transplant (SOT), and 29 (2%) were taking daily steroids (>10 mg of prednisone daily or an equivalent). As shown by radiological imaging, 1238/1460 (84%) had signs of pneumonia at the time of admission. During hospitalization, 328 (25.8%) patients developed moderate or severe ARDS. The incidence of ARDS was higher in those with pneumonia at admission, (296/1238, 29.1%) than in the rest of the cohort (26/222, 11.7%).

### 3.2. Mortality and Risk Factors

A total of 422 (27%) patients passed away during hospitalization. The median age of the deceased patients was 83 years (IQR 76–87); the median age of the patients who were discharged was 69 years (IQR 57–80) and over 88% of the deceased patients were >70 years old.

In the hospital, the death rate was higher in patients with comorbidities and in those admitted with fever, respiratory or gastrointestinal symptoms and was strongly associated with lower PaO_2_/FiO_2_ (P/F) ratios. Patients who died in hospital had a significantly shorter time from symptom onset to positive test (3 vs. 5 days) and hospitalization (3 vs. 7 days).

Baseline laboratory data and therapies are reported in Table 2. The deceased patient showed lower values of total lymphocytes and eGFR and higher values for LDH, D-dimer, CRP and PCT.

The majority of patients received antibiotics (81%), hydroxychloroquine (67%) or antivirals (62%). LMWH was administered to 45%, steroids to 26% and only a few patients received tocilizumab (7%) or RDV (1%).

As many as 76% of patients received oxygen supplementation during hospitalization, 25% of patients underwent non-invasive ventilation, and 8% later required mechanical ventilation. All respiratory supports were more often employed in patients at higher risk of in-hospital mortality.

The role of patients’ characteristics at admission on hospital mortality was analyzed with a logistic regression model, including all variables reported in Table 3. Both unadjusted and adjusted analyses confirmed a positive association with in-hospital mortality for increasing age and some comorbidities (diabetes, cardiovascular disease, immunosuppression) but not for hypertension. No association was detected for sex and smoking (ever vs. never). As expected, a clear trend of reduced risk of mortality was associated with higher PaO_2_/FiO_2_ ratios.

As lymphocytopenia, elevated LDH and D-dimer, some laboratory values positively associated with mortality in unadjusted analyses were not confirmed when adjusted for the other variables. A positive association was confirmed for elevated CRP values and reduced eGFR.

To examine the connection between treatments and mortality, patients who died shortly after admission and those discharged alive after a few days of hospitalization were excluded because of early recovery. Another reason for analyzing only those patients who were alive at day 7 was to avoid a survival time bias (the recording of some drugs was conditioned on a minimum of 5 days of treatment). The drugs employed in the subgroup of patients alive at day 7 are described in Table 4. Overall, this subgroup’s mortality rate was lower than in the total cohort (19.6% vs. 27%). Patients treated with some antivirals or with HCQ showed a lower risk of death in this unadjusted comparison.

The associations between drug treatments and mortality are reported in Table 5, as unadjusted and adjusted ORs. Hydroxychloroquine is the only treatment confirming a strong association with reduced mortality (aOR: 0.57; 95% CI: 0.36–0.90, *p* = 0.015), despite careful adjustment for a large set of potential confounders. Sensitivity analyses were conducted to determine this finding’s robustness by using a propensity score approach, either as a model covariate (aOR: 0.66) or using an inverse probability of treatment weight (IPTW, aOR:0.60), confirming a statistically significant protective effect with both methods. Lastly, to exclude a selection effect of the analyzed population alive at day 7 (*n* = 1005), we re-estimated the adjusted effect of HCQ also on the total population (*n* = 1527, aOR: 0.51) and those alive at day 3 (*n* = 1300, aOR: 0.61). All the results of these sensitive analyses confirmed the clear negative association between HCQ and hospital mortality.

## 4. Discussion

The CORACLE Registry is an experience of a regional infectious disease network during the first “wave” of the COVID-19 pandemic in Piedmont, Italy. Comparable and similar methodologies have been used in European and non-European countries recently [20,21]. In Spain, the Spanish Society of Internal Medicine (SEMI) has collected clinical features from 15,111 patients for the SEMI-COVID-19 national registry through 150 hospitals [20]. The Spanish epidemiology of SARS-CoV-2 infection is highly similar to its Italian counterpart. At the time of writing, Italy and Spain’s total confirmed cases (at 23 December, 1,990,000 vs. 1,842,000, respectively) and deaths (70,000 vs. 49,000, respectively) are analogous and thus suitable for comparison [6]. The proportion of males in the CORACLE and the Spanish national registry [20] is homogeneous (58% vs. 57.2%), but the median age in the Piedmont cohort is higher than that in the Spanish one (74 vs. 69.4 years). This difference alone may explain the higher mortality in our registry (27% vs. 21%). The association of mortality with older age is a prominent feature of COVID-19: 88% of our report’s deaths are adults aged >70 years, confirming all previous data [21].

Senior patients may have a less vigorous immune response due to immunosenescence [22] and more comorbidities; these combined factors may explain the higher risk of death of older patients. Contrary to previous findings [23,24,25], the present cohort showed the same risk of in–hospital mortality for males and females. The most common comorbidities between CORACLE and SEMI-COVID-19 are hypertension (49% vs. 50.9%, respectively) and diabetes mellitus (21% vs. 19.4%, respectively) [20]. Being a smoker (both current and former) is not connected with higher or lower mortality in multivariate analysis [26]. Many studies have found that being a former smoker is associated with a higher fatality risk, whereas being an active smoker is not [27]. This association is not found in the present study, although the low prevalence of active smokers among COVID-19 patients has not been wholly explained [27]. Smokers compared to non-smokers present an upregulation of pulmonary ACE2 (angiotensin-converting enzyme 2) gene expression, the well-known receptor used by SARS-CoV-2 to enter the host cells [28,29,30,31].

Furthermore, smoking tobacco causes alterations of the bronchial epithelium with lower protective bronchial mucous [32]. Cai et al. [32] indicated that the overall lower-than-expected smoking prevalence reported in retrospective and observational datasets was likely due to a lack or inaccuracy of smoking trend knowledge. Finally, structural changes in smoke-related inflammation-induced ACE2 allelic variants can interfere with the intermolecular interactions of such variants with SARS-CoV-2 spike protein and the smoker’s respiratory dynamics can hold away the droplets during cigarette smoke intake and exhalation. Interestingly, smoke-related lung comorbidities, such as COPD, have a higher prevalence in the CORACLE registry (11.4% vs. 6.9%), but asthma prevails in the SEMI-COVID-–19 cohort (1.5% vs. 7.3%) [20]. A recent meta-analysis by Zhao et al. recorded a pooled OR of COPD and the development of severe COVID-19 of 4.38 (95% CI: 2.34–8.20) [33].

Increased morbidity and mortality among COPD subjects infected with SARS-CoV-2 may be associated with insufficient underlying lung reserves or higher ACE2 receptor expression in small airways, according to Leung et al. [34]. In addition, according to Kumar et al. [35], the combined corrected pooled OR of mortality of diabetes is 2.16 (95% CI: 1.74–2.68, *p* < 0.01), which is higher than CORACLE’s related mortality (aOR = 1.42 [95% CI: 1.03–1.96, *p* = 0.032]. Comments are consistent with compromised innate immunity, the first line of defense against SARS-CoV-2. Diabetic patients may suffer from chronic inflammation or increased coagulation activity, raising the risk of worse performance [36,37].

Severe renal impairment with eGFR ≤ 30 mL/min/1.73 m^2^ and lymphocytopenia at admission (even if to a lesser degree after adjustment) was associated with higher mortality. Lymphopenia is a useful and reliable indicator of the severity and hospitalization in COVID-19 patients [38], although, in our data, it does not seem utterly independent from other prognostic factors.

Absolute lymphopenia is the main feature of severe SARS-CoV-2 infection and is a significant feature of clinical phases immediately before the deterioration in respiratory functions and the need for oxygen or added ventilation [39]. Homing phenomena or chemotaxis may sign incipient interstitial lymphocytic pneumonia to explain the lymphopenia’s potential pathogenic meaning better [40]. The early-stage lymphocytic alveoli or interstitial patterns are observed in post-mortem biopsies in COVID-19 subjects, followed by acute fibrinous organizational pneumonia which culminates in diffuse alveolar harm [38,40,41]. Interestingly, lung inflammation increased after viral clearance in SARS animal models and reached its maximum point about two weeks after infection [39,40]. In SARS patients, similar observations have been made as to whether uncontrolled viral replication or uncontrolled immune responses may cause injury [41,42,43].

Higher mortality associated with renal failure was also showed by GECOVID working group in which the mortality was 63% (111/176 patients) in acute kidney injury (both new onset or worsening of pre-existing CKD), and its occurrence increased the risk of fatality by 60% (HR 1.60 (95% IC 1.21–2.49) *p* = 0.002) [44].

In our cohort, fever, cough, dyspnea and asthenia are the most common of the symptoms that were reported after admission [45,46,47]. Without any lung involvement, people were admitted only in the most important cases to external hospitals from emergency departments.

Anti-SARS-CoV-2 regimens’ efficiency and safety are still greatly debated. Among the treatments that exhibit theoretical anti-SARS-CoV-2 efficacy, HCQ is prevalent in both CORACLE and SEMI-COVID-19, but more frequently prescribed in Spain hospitals (67% vs. 85.6%, respectively), followed by LPV/r (24.5% vs. 61.4%). Furthermore, tocilizumab (7% vs. 8.5%, respectively) and systemic steroids (26% vs. 35.2%, respectively) were less used in our patients.

All these treatments were not cleared for appropriate use during the first wave and, specifically, HCQ was highly debated owing to early results of observational, poor-quality studies. Cavalcanti et al. [48] recently published the randomized tri-group, multi-center trial open-label on potential or validated COVID-19 hospital patients who have not been given, or up to 4 L/min of, extra oxygen. In a recent study published, 667 persons did not boost their clinical status in a mild- to medium-size COVID-19 series, using HCQ alone or with azithromycin on day 15 [49]. Moreover, a complete systematic analysis by Hong et al. stated that HCQ used by itself or combined with other drugs did not significantly lower deaths in hospitalized patients with COVID-19 (OR: 0.95 95% CI: 0.72–1.26, *p* = 0.732, I_2_ = 91.05). However, compared to the current study, the inclusion criteria of several series included by Hong et al. were clinical and confirmed molecular diagnoses of SARS-CoV-2 infection [50]. Despite the fact that the role of HCQ in the treatment of COVID-19 pneumonia is still controversial and some studies evidenced a positive effect on mortality reduction [51,52], the result of the largest randomized trial did not suggest any beneficial effect of HCQ [53]. In our study, despite careful adjustment for several potential confounders and sensitivity analyses conducted with different methods and patient selections, HCQ was considerably connected to lower death rates in patients affected by COVID-19 pneumonia. Nonetheless, due to the CORACLE registry’s observation design, this result should be considered with great caution.

This study has a variety of limitations caused by the exquisite registry characteristics, such as the retrospective collection of data, the exclusion of patients directly requiring critical care admission, the dynamic weekly evolution of the different treatment patterns and the progressive global reduction of available treatment with the publications of new data. Centers involved in the study belong to the PIDUN network; therefore, there was a shared management of COVID-19 patients. Of course, due to the retrospective nature of the study, data have been reviewed and collected upon request for the CORACLE registry; a few of them may not be available for all patients, but the numbers were low and did not affect the statistical analysis. Even if there is a statistically significant association of HCQ with lower mortality, this result may be due to unmeasured or residual confounders, or a collider bias in selecting enrolled patients [54]. Finally, although the present work did adjust the data for severity, the different approaches used at the different centers could have introduced sources of heterogeneity in patient selection and treatment.

## 5. Conclusions

The CORACLE Registry confirmed the negative prognostic role of older age, cardiovascular diseases, COPD, immunosuppression, diabetes, patients presenting with an eGFR < 30 mL/min/1.73 m^2^, high values of CRP and a low PaO_2_/FiO_2_ ratio upon admission of COVID-19 patients on hospital mortality. The CORACLE registry may be useful for comparing similar, real-world, regional or national databases and monitoring modifications of patients’ characteristics, treatments, and outcomes during this time. The finding that only HCQ was heavily connected to lower death rates should be interpreted with great caution, and further studies should carefully be evaluated.

## Figures and Tables

**Table 1:** Demographic and clinical characteristics of enrolled population at baseline.

	Total *n* = 1538	In-Hospital Mortality, *n* (%)		*p*-Value
Variables [Number of Available Data]	*n* (%)	Yes *n* = 422 (27%)	No *n* = 1116 (73%)	
Sex (1533), *n* (%):				0.448
− F	641 (42%)	183 (29%)	458 (71%)	
− M	892 (58%)	239 (27%)	653 (73%)	
Age (1538), median (IQR):	74 (61–83)	83 (76–87)	69 (57–80)	<0.001
Age distribution (1538), *n* (%):				<0.001
- ≤50 y	153 (10%)	4 (3%)	149 (97%)	
- 51–70 y	490 (32%)	48 (10%)	442 (90%)	
- 71–80 y	379 (24%)	116 (31%)	263 (69%)	
- 81–90 y	413 (27%)	197 (48%)	216 (52%)	
- >90 y	103 (7%)	57 (55%)	46 (45%)	
Smokers (1121), *n* (%):				0.033
- active	76 (7%)	13 (17%)	63 (83%)	
- former	256 (23%)	83 (32%)	173 (68%)	
- never smoked	789 (70%)	223 (28%)	566 (72%)	
Comorbidities (1538), *n* (%):				
- Diabetes	324 (21%)	120 (37%)	204 (63%)	<0.001
- Hypertension	759 (49%)	250 (33%)	509 (67%)	<0.001
- Dementia	309 (20%)	161 (52%)	148 (48%)	<0.001
- Cardiovascular diseases	490 (32%)	218 (44%)	272 (56%)	<0.001
- Lung diseases				<0.001
- COPD	175 (11.4%)	84 (48%)	91 (52%)	
- Asthma	23 (1.5%)	5 (22%)	18 (78%)	
- Other	1 (0.1%)	1 (100%)	0 (0%)	
Immunosuppression (1538), *n* (%):	148 (10%)	53 (36%)	95 (64%)	0.016
- Active solid tumors	74 (5%)	31 (42%)	43 (58%)	0.004
- Blood cancers	38 (2%)	12 (32%)	26 (68%)	0.558
- Immunosuppressive therapies, Transplanted patients	21 (1%)	5 (24%)	16 (76%)	0.711
- Chronic steroidal use	29 (2%)	9 (31%)	20 (69%)	0.657
- HIV	1 (0.1%)	0 (0%)	1 (100%)	0.539
Symptoms at admission, *n* (%):	1249 (93%)	353 (28%)	896 (72%)	0.758
- fever (1346)	1023 (76%)	270 (26%)	753 (74%)	0.005
- dyspnea (1346)	719 (53%)	250 (35%)	469 (65%)	<0.001
- myalgia (1346)	221 (16%)	40 (18%)	181 (82%)	<0.001
- sore throat (848)	23 (3%)	4 (17%)	19 (83%)	0.152
- cough (1346)	573 (43%)	105 (18%)	468 (82%)	<0.001
- diarrhea (1346)	172 (13%)	27 (16%)	145 (84%)	<0.001
- no symptoms (1346)	97 (7%)	26 (27%)	71 (73%)	0.758
Days from symptom onset to positive test (1248), median (IQR):	4 (1–8)	3 (0–6)	5 (2–9)	<0.001
Days from symptoms onset to hospital admission (1445), median (IQR):	6 (2–10)	3 (0–7)	7 (3–10)	<0.001
PaO_2_/FiO_2_ at admission (distribution) (1019), *n* (%):				<0.001
- <200	180 (18%)	114 (63%)	66 (37%)	
- 200–299	412 (40%)	150 (36%)	262 (64%)	
- ≥ 300	427 (42%)	68 (16%)	359 (84%)	
Pneumonia (1469), *n* (%):	1238 (84%)	355 (29%)	883 (71%)	0.074

COPD, chronic obstructive pulmonary disease; IQR, Inter Quartile Range; F, female; M, male; HIV, Human Immunodeficiency Virus.

**Table 2:** Laboratory data at baseline and treatments of enrolled population.

	Total *n* = 1538	In-Hospital Mortality, *n* (%)		*p*-Value
Variables [Number of Available Data]	*n* (%)	Yes *n* = 422 (27%)	No *n* = 1116 (73%)	
% Lymphocytes (1312), median (IQR):	15 (8.9–22.1)	10.5 (6.7–18.4)	16.5 (10.1–23)	<0.001
Lymphopenia (<1000) (1312), *n* (%):	694 (53%)	231 (33%)	463 (67%)	<0.001
LDH (U/L) (1200), median (IQR):	582 (436–766)	699 (520–900)	552 (422–713)	<0.001
D-dimer (ng/mL) (800), median (IQR):	1200 (610–2290)	1825 (966–3325)	959 (550–1875)	<0.001
CRP (mg/L) (1461), median (IQR):	74 (28–139)	109 (52–175)	62 (21–124)	<0.001
PCT (ng/mL) (904), median (IQR):	0.1 (0.1–0.4)	0.3 (0.1–0.9)	0.1 (0.1–0.2)	<0.001
PCT (distribution) (904), *n* (%):				<0.001
- ≤0.08	248 (27%)	33 (13%)	215 (87%)	
- 0.08–0.14	213 (24%)	50 (23%)	163 (77%)	
- 0.14–0.39	218 (24%)	74 (34%)	144 (66%)	
- >0.39	225 (25%)	120 (53%)	105 (47%)	
eGFR (1282), median (IQR)	77.2 (48.4–96.0)	50.4 (28.4–79.1)	83.9 (61.8–100.3)	<0.001
EGFR (mL/min/1.73 m^2^) (1282), *n* (%):				<0.001
- >60	852 (67%)	150 (18%)	702 (82%)	
- 30–60	274 (21%)	117 (43%)	157 (57%)	
- ≤30	156 (12%)	97 (62%)	59 (38%)	
ESRD (1282), *n* (%):	63 (5%)	37 (59%)	26 (41%)	<0.001
LMWH (1531), *n* (%):	690 (45%)	212 (31%)	478 (69%)	0.008
Antibiotics (1502), *n* (%):	1221 (81%)	351 (29%)	870 (71%)	0.242
Steroids (1454), *n* (%):	381 (26%)	82 (22%)	299 (78%)	0.001
Type of steroids (1454), *n* (%):				0.002
- methylprednisolone	171 (12%)	45 (26%)	126 (74%)	
- dexamethasone	120 (8%)	26 (22%)	94 (78%)	
- other	15 (1%)	0 (0%)	15 (100%)	
- not specified	75 (5%)	11 (15%)	64 (85%)	
Antivirals (1525), *n* (%):				0.001
- LPV/r	373 (25%)	84 (23%)	289 (77%)	
- DRV/r	14 (1%)	2 (14%)	12 (86%)	
- DRV/c	182 (12%)	34 (19%)	148 (81%)	
Remdesivir (1335), *n* (%):	7 (1%)	1 (14%)	6 (86%)	0.405
Hydroxychloroquine (1527), *n* (%):	1019 (67%)	207 (20%)	812 (80%)	<0.001
Tocilizumab (1336), *n* (%):	97 (7%)	15 (15%)	82 (85%)	0.004
Oxygen therapy (1500), *n* (%):	1135 (76%)	381 (34%)	754 (66%)	<0.001
- CPAP, NIV, HFNC (1477)	370 (25%)	95 (26%)	275 (74%)	0.569
- Mechanical ventilation (1500)	116 (8%)	23 (20%)	93 (80%)	0.049
Oxygen therapy at discharge (693), *n* (%):	69 (10%)	-	69 (100%)	-
Days of hospitalization (1487), median, *n* (%):	10 (5–18)	6 (2–12)	12 (7–20)	<0.001

LDH, Lactate dehydrogenase; CRP, C-reactive protein; PCT, procalcitonin; eGFR, estimated glomerular filtration rate; ESRD, end stage renal disease, defined by EGFR< 15 mL/min/1.73 m^2^; LMWH, low molecular weight heparin, given for at least 72 h; antibiotics, administration of antibiotics at admission; steroids, administration of steroids for at least 5 days; LPV/r, lopinavir/ritonavir; DRV/r, darunavir/ritonavir; DRV/c, darunavir/cobicistat; CPAP, continuous positive airway pressure; NIV, non-invasive ventilation; HFNC, high flow nasal cannula.

**Table 3:** Association between demographic and clinical characteristics of patients in the total cohort (*n* = 1538) and in-hospital mortality. Raw and adjusted Odds Ratios (OR) and 95% CI estimated with a logistic regression model.

	OR (95% CI) (Univariate Model)	*p*-Value	OR (95% CI) (Multivariate Model) *	*p*-Value
Sex (M vs. F)	0.92 (0.73–1.15)	0.448	1.13 (0.84–1.53)	0.417
Age at admission (per year)	1.09 (1.08–1.10)	<0.001	1.07 (1.06–1.09)	<0.001
Smoking				
- active vs. never smoked	0.52 (0.28–0.97)	0.040	0.99 (0.47–2.13)	0.996
- former vs. never smoked	1.22 (0.90–1.65)	0.204	1.31 (0.88–1.95)	0.177
Comorbidities (present vs. not present):				
- Diabetes	1.78 (1.37–2.31)	<0.001	1.41 (1.02–1.94)	0.038
- Hypertension	1.74 (1.39–2.19)	<0.001	0.78 (0.58–1.05)	0.098
- Cardiovascular diseases	3.33 (2.63–4.21)	<0.001	1.79 (1.31–2.44)	<0.001
- COPD	2.81 (2.03–3.87)	<0.001	1.48 (0.99–2.20)	0.056
- Asthma	0.84 (0.31–2.29)	0.740	1.45 (0.44–4.78)	0.546
- Immunodepression	1.55 (1.08–2.21)	0.016	1.65 (1.04–2.62)	0.034
Characteristics at admission:				
P/F:				
- 200–300 vs. <200	0.33 (0.23–0.48)	<0.001	0.41 (0.27–0.65)	<0.001
- ≥300 vs. <200	0.11 (0.07–0.16)	<0.001	0.22 (0.13–0.36)	<0.001
Lymphocytopenia (yes vs. no)	1.70 (1.33–2.18)	<0.001	1.28 (0.94–1.76)	0.120
LDH (U/L):				
- 437–582 vs. ≤436	1.19 (0.79–1.79)	0.406	0.81 (0.49–1.33)	0.405
- 583–766 vs. ≤436	1.80 (1.22–2.66)	0.003	1.22 (0.75–1.99)	0.427
- >766 vs. ≤436	3.41 (2.34–4.96)	<0.001	1.60 (0.97–2.62)	0.065
D-dimer (ng/mL):				
- 611–1200 vs. ≤610	1.70 (1.01–2.84)	0.046	1.02 (0.55–1.89)	0.943
- 1201–2290 vs. ≤610	2.97 (1.80–4.90)	<0.001	1.01 (0.55–1.86)	0.980
- >2290 vs. ≤610	5.00 (3.07–8.14)	<0.001	1.44 (0.78–2.65)	0.241
CRP (mg/L):				
- 28–74 vs. ≤28	2.55 (1.74–3.74)	<0.001	1.75 (1.11–2.74)	0.015
- 74–139 vs. ≤28	2.80 (1.92–4.10)	<0.001	1.49 (0.94–2.37)	0.089
- >139 vs. ≤28	5.17 (3.57–7.49)	<0.001	2.17 (1.36–3.45)	0.001
eGFR (mL/min/1.73 m^2^):				
- 30–60 vs. >60	3.49 (2.59–4.70)	<0.001	1.47 (1.03–2.11)	0.034
- ≤30 vs. >60	7.69 (5.32–11.12)	<0.001	3.53 (2.26–5.51)	<0.001

COPD, chronic obstructive pulmonary disease; P/F, PaO_2_/FiO_2_ ratio; LDH lactate dehydrogenase; CRP, C-reactive protein; eGFR, estimated glomerular filtration rate. * All the variables in the table are used for adjustment in the multivariate model.

**Table 4:** Distribution of treatments administered in patients alive at day 7 after admission.

	Total *n* = 1011	In–Hospital Mortality, *n* (%)		*p*-Value
Variables [Number of Available Data]	*n* (%)	Yes *n* = 198 (20%)	No *n* = 813 (80%)	
LMWH (1008)	500 (50%)	107 (21%)	393 (79%)	0.140
Steroids (970)	305 (31%)	62 (20%)	243 (80%)	0.777
Antivirals (1007)	408 (41%)	66 (16%)	342 (84%)	0.022
Lopinavir/Ritonavir (1007)	251 (25%)	40 (16%)	211 (84%)	0.086
Darunavir /Ritonavir or Cobicistat (1007)	158 (16%)	26 (16%)	132 (84%)	0.269
Remdesivir (882)	7 (1%)	1 (14%)	6 (86%)	0.701
Hydroxychloroquine (1008)	731 (73%)	118 (16%)	613 (84%)	<0.001
Tocilizumab (884)	90 (10%)	13 (14%)	77 (86%)	0.196

LMWH, low molecular weight heparin, given for at least 72 h.

**Table 5:** Association of drug treatments with hospital mortality in 1005 patients alive at day 7 after admission.

Drug treatments (Yes vs. No)	OR (95% CI) (Univariate)	*p*-Value	OR (95%CI) (Adjusted) *	*p*-Value
LMWH	1.26 (0.92–1.72)	0.151	1.13 (0.72–1.77)	0.597
Steroids	1.07 (0.76–1.50)	0.696	0.91 (0.58–1.43)	0.692
Lopinavir/Ritonavir	0.72 (0.49–1.06)	0.094	1.07 (0.60–1.89)	0.818
Darunavir /Ritonavir or Cobicistat	0.78 (0.50–1.23)	0.282	1.39 (0.75–2.57)	0.295
Hydroxichloroquine	0.48 (0.35–0.67)	<0.001	0.57 (0.36–0.90)	0.015
Tocilizumab	0.67 (0.37–1.24)	0.201	1.41 (0.66–2.99)	0.377

* OR estimated with a logistic regression model adjusted for age, sex, smoking, diabetes, hypertension, cardiovascular disease, lung disease, immunosuppression, PO_2_/FIO_2_ ratio, *n*° Lymphocytes, LDH, D-dimer, CRP, eGFR.

## Data Availability

The data presented in this study are available on request from the corresponding author.
